# Attention Deficit/Hyperactivity Disorder and Childhood Autism in Association with Prenatal Exposure to Perfluoroalkyl Substances: A Nested Case–Control Study in the Danish National Birth Cohort

**DOI:** 10.1289/ehp.1408412

**Published:** 2014-12-19

**Authors:** Zeyan Liew, Beate Ritz, Ondine S. von Ehrenstein, Bodil Hammer Bech, Ellen Aagaard Nohr, Chunyuan Fei, Rossana Bossi, Tine Brink Henriksen, Eva Cecilie Bonefeld-Jørgensen, Jørn Olsen

**Affiliations:** 1 Department of Epidemiology, Fielding School of Public Health, University of California, Los Angeles, Los Angeles, California, USA; 2 Department of Neurology, School of Medicine, University of California, Los Angeles, Los Angeles, California, USA; 3 Department of Community Health Sciences, Fielding School of Public Health, University of California, Los Angeles, Los Angeles, California, USA; 4 Section for Epidemiology, Department of Public Health, University of Aarhus, Aarhus, Denmark; 5 Department of Gynaecology and Obstetrics, Odense University Hospital, Odense, Denmark; 6 Global Surveillance & Pharmacoepidemiology, AbbVie Inc., North Chicago, Illinois, USA; 7 Department of Environmental Science, University of Aarhus, Roskilde, Denmark; 8 Perinatal Epidemiology Research Unit, Department of Paediatrics, Aarhus University Hospital, Skejby, Denmark; 9 Centre for Arctic Health and Unit of Cellular and Molecular Toxicology, Department of Public Health, Aarhus University, Aarhus, Denmark

## Abstract

**Background:**

Perfluoroalkyl substances (PFASs) are persistent pollutants found to be endocrine disruptive and neurotoxic in animals. Positive correlations between PFASs and neurobehavioral problems in children were reported in cross-sectional data, but findings from prospective studies are limited.

**Objectives:**

We investigated whether prenatal exposure to PFASs is associated with attention deficit/hyperactivity disorder (ADHD) or childhood autism in children.

**Methods:**

Among 83,389 mother–child pairs enrolled in the Danish National Birth Cohort during 1996–2002, we identified 890 ADHD cases and 301 childhood autism cases from the Danish National Hospital Registry and the Danish Psychiatric Central Registry. From this cohort, we randomly selected 220 cases each of ADHD and autism, and we also randomly selected 550 controls frequency matched by child’s sex. Sixteen PFASs were measured in maternal plasma collected in early or mid-pregnancy. We calculated risk ratios (RRs) using generalized linear models, taking into account sampling weights.

**Results:**

Perfluorooctane sulfonate (PFOS) and perfluorooctanoic acid (PFOA) were detected in all samples; four other PFASs were quantified in ≥ 90% of the samples. We did not find consistent evidence of associations between mother’s PFAS plasma levels and ADHD [per natural log nanograms per milliliter increase: PFOS RR = 0.87 (95% CI: 0.74, 1.02); PFOA RR = 0.98 (95% CI: 0.82, 1.16)] or autism [per natural log nanograms per milliliter increase: PFOS RR = 0.92 (95% CI: 0.69, 1.22); PFOA RR = 0.98 (95% CI: 0.73, 1.31)]. We found positive as well as negative associations between higher PFAS quartiles and ADHD in models that simultaneously adjusted for all PFASs, but these estimates were imprecise.

**Conclusions:**

In this study we found no consistent evidence to suggest that prenatal PFAS exposure increases the risk of ADHD or childhood autism in children.

**Citation:**

Liew Z, Ritz B, von Ehrenstein OS, Bech BH, Nohr EA, Fei CY, Bossi R, Henriksen TB, Bonefeld-Jørgensen EC, Olsen J. 2015. Attention deficit/hyperactivity disorder and childhood autism in association with prenatal exposure to perfluoroalkyl substances: a nested case–control study in the Danish National Birth Cohort. Environ Health Perspect 123:367–373; http://dx.doi.org/10.1289/ehp.1408412

## Introduction

Perfluoroalkyl substances (PFASs) are a group of human-made fluorine-containing compounds with unique properties that make materials resistant to stains, oil, and water (Buck et al. 2011). PFASs have been used widely in commercial products since the 1950s, and they are persistent in the environment and in living organisms throughout the globe (Houde et al. 2006). Human exposure routes include contamination of food from packaging, bioaccumulation in the food chain, and household dust (D’eon and Mabury 2011). Perfluorooctane sulfonate (PFOS) and perfluorooctanoic acid (PFOA) are the two most frequently used PFASs; they have estimated biological half-lives in humans between 4 and 5 years (Olsen et al. 2007). PFOS and PFOA concentrations in humans were reported to be decreasing in some countries following a drop in production since 2000 (Kato et al. 2011), but exposure to other short-chain compounds such as perfluorobutane sulfonate (PFBS) and perfluorohexane sulfonate (PFHxS) and long-chain perfluorononanoic acid (PFNA) and perfluorodecanoic acid (PFDA) are reported to be increasing (Glynn et al. 2012).

PFASs can cross the placental barrier and expose the fetus during the most vulnerable period of development (Fei et al. 2007). Experimental data suggest that PFASs may be developmental neurotoxicants that can affect neuronal cell development (Slotkin et al. 2008), alter cognitive function, and reduce habituation and learning ability in mice (Johansson et al. 2008, 2009; Viberg et al. 2013). PFASs also have endocrine-disruptive properties (Kjeldsen and Bonefeld-Jørgensen 2013) and might interfere with thyroid hormone function (Lau et al. 2003; Lin et al. 2013; Long et al. 2013; Wang et al. 2014), which is essential in regulating fetal brain development (Porterfield 2000).

Attention deficit/hyperactivity disorder (ADHD) is considered one of the most common neurobehavioral disorders worldwide, and is characterized by inattention, hyperactivity, increased impulsivity, and motivational/emotional dysregulation (Polanczyk et al. 2007). Autism is a neurodevelopmental disorder characterized by impairments in communication and reciprocal social interaction, coupled with repetitive behavior (Pickett and London 2005). The incidence of ADHD and autism has increased over the past decades, and it has been suggested that the rise is not attributable solely to changes in diagnostic practices or parental awareness (Faraone et al. 2003; Hertz-Picciotto and Delwiche 2009; Møller et al. 2007). The etiologies are not well understood, but both environmental and genetic factors are thought to contribute to ADHD and autism (Lyall et al. 2014; Millichap 2008). ADHD and autism disproportionately affect boys (Arnold 1996), and studies suggest that prenatal exposure to endocrine-disrupting chemicals may be associated with the occurrence of both diseases (de Cock et al. 2012).

A limited number of epidemiologic studies have evaluated the potential neurobehavioral or neurocognitive impact of PFASs and findings were inconclusive. Several cross-sectional studies have reported positive associations between serum levels of some PFASs with impulsivity (Gump et al. 2011) and ADHD in children (Hoffman et al. 2010; Stein and Savitz 2011). Reverse causality, however, is a concern for studies that measure PFAS levels in children already diagnosed with ADHD at time of blood draw. Little evidence of associations was found for prenatal exposures to PFOS or PFOA and behavioral problems in 7-year-old children assessed with the Strengths and Difficulties Questionnaires in the prospective Danish birth cohort (Fei and Olsen 2011). A study conducted in a community with high long-term exposure to PFOA in contaminated drinking water reported that *in utero* PFOA levels were associated with higher Full-Scale IQ and decreased ADHD characteristics among children 6–12 years of age (Stein et al. 2013). However, prenatal PFOA exposures were estimated based on exposure modeling. A recent study examined the associations between several endocrine-disrupting chemicals, including PFASs, and autistic behaviors in children but no conclusive evidence was found, perhaps due to small sample size (175 mother–child pairs) and low statistical power (Braun et al. 2014).

We conducted a nested case–control study within the framework of the Danish National Birth Cohort (DNBC) to examine whether prenatal exposure to PFASs is associated with ADHD or autism in children.

## Methods

The DNBC is a nationwide cohort study of pregnancies and health-related outcomes in the children; details have been described elsewhere (Olsen et al. 2001). Briefly, pregnant women were recruited through their general practitioners during early gestation (weeks 6–12) from 1996 to 2002. About 50% of all general practitioners in Denmark participated in the study, and 60% of the women invited agreed to participate. Women were ineligible if they did not speak sufficient Danish for interviews or intended not to carry their pregnancy to term. Information was collected during four computer-assisted telephone interviews (twice during pregnancy and twice postpartum). Two prenatal maternal blood samples were collected and stored, one each in the first and second trimester. English versions of questionnaires are available online (Statens Serum Institut 2013).

Written informed consent was obtained from all participants at recruitment. Study procedures have been approved by the Danish Data Protection Agency and the Institutional Review Board at University of California, Los Angeles.

*Source population*. The source population for this study consisted of live-born singletons along with mothers who participated in the first telephone interview, conducted approximately during the 12th gestational week, and had provided a blood sample at least once either during the first or second pregnancy trimesters. This resulted in 83,389 mother–child pairs, with 42,737 boys and 40,652 girls; we excluded from the original DNBC those with an unsuccessful pregnancy (*n* = 6,207), non-singleton births (*n* = 2,080), births with unknown birth outcomes (*n* = 25) or missing dates of birth (*n* = 99), mothers who emigrated (*n* = 51) or died (*n* = 3), and women who did not participate in the first telephone interview (*n* = 4,578) or did not provide a prenatal blood sample (*n* = 4,609).

*Selection of cases and controls*. We identified children who were diagnosed with ADHD and autism, respectively, by linking DNBC records to the Danish National Hospital Registry (Andersen et al. 1999) that contains the nationwide data for all admissions for somatic illnesses, and also to the Danish Psychiatric Central Registry (Munk-Jørgensen et al. 1993), which covers admissions to all psychiatric hospitals in Denmark. The record linkage relied on the unique civil registration numbers given to all Danish citizens at birth. All diagnoses are based on the *International Classification of Diseases, 10th Revision* (ICD-10 codes F90.0 for ADHD and F84.0 for childhood autism) and included inpatient and outpatient records. A total of 890 ADHD cases and 301 autism cases were identified in the cohort during an average of 10.7 years of follow-up (record linkage was conducted on 1 August 2011). Because of the high costs of measuring PFASs, we randomly selected 220 cases of ADHD and autism each for inclusion in this study.

**Figure 1:**
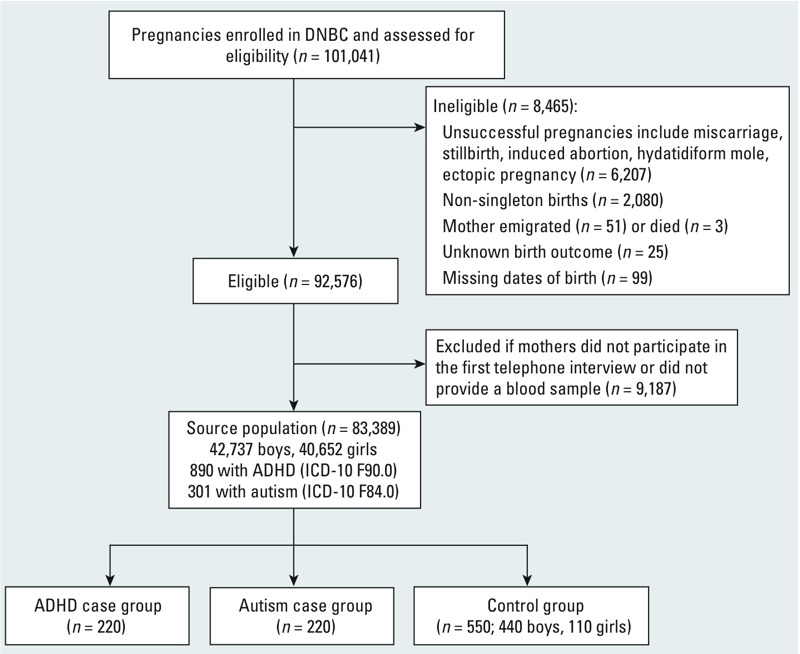
Flow chart of study population selection in the Danish National Birth Cohort. Sampling fraction of ADHD cases is 0.2472. Sampling fraction of autism cases is 0.7309. Sampling fractions of control group are 0.0103 for male and 0.0027 for female.

We used a case-cohort sampling strategy and randomly selected 550 children (440 males and 110 females) as controls from the source population, frequency matched to cases by sex. The flow chart of subject selection and sampling fractions of cases and controls is shown in Figure 1.

*PFAS measurements*. Details about analytic methods for PFASs have been described previously (Liew et al. 2014). Briefly, the collected maternal blood samples were sent by mail to Statens Serum Institut in Copenhagen, and separated and stored in freezers at –20°C or –80°C or in liquid nitrogen. We used 0.1 mL stored maternal plasma, and the samples were analyzed at the Department of Environmental Science at Aarhus University. Most of the samples (87%) for both cases and controls were collected during the first trimester; if the first sample was not available, we used the second sample collected in the second trimester instead. Solid-phase extraction technique was used for extraction and purification, and PFAS concentrations were measured by liquid chromatography–tandem mass spectrometry. Measurements were performed in a random sequence for cases and controls by laboratory personnel blinded to diagnoses and any other participant information. Seventeen maternal samples (5 ADHD, 7 autism, 5 controls) were either not available from the biobank or failed the PFAS extraction and purification process, and hence were excluded. For 21 samples included in our current study, PFOA and PFOS values previously had been analyzed at the 3M Toxicology Laboratory for an earlier study in the DNBC (Fei et al. 2007). For quality control we compared the PFOA and PFOS value measured from the two laboratories in these samples and found high correlations (Pearson *r* = 0.94 for PFOS and *r* = 0.95 for PFOA).

Of the 16 different PFASs detected in maternal plasma, we focused on the six PFASs for which at least 90% of all samples were above the lower limit of quantitation (LLOQ): PFOS, 100%; PFOA, 100%; PFHxS, 98%; PFHpS (perfluoroheptane sulfonate), 96%; PFNA, 92%; and PFDA, 90%. The LLOQ for the six PFASs are as follows: PFOS, 0.28 ng/mL; PFOA, 0.20 ng/mL; PFHxS, 0.08 ng/mL; PFHpS, 0.11 ng/mL; PFNA, 0.27 ng/mL; and PFDA, 0.09 ng/mL. The full panel for the LLOQ and distribution of all PFASs was reported previously (Liew et al. 2014).

*Statistical analysis*. We used generalized linear models and accounted for the sampling fractions of cases and controls to estimate risk ratios (RR) and 95% confidence intervals (CIs) for prenatal PFAS exposures and ADHD or autism. We also performed unconditional logistic regression to estimate odds ratios (ORs) without applying the sampling weights. PFAS concentrations were first analyzed as continuous variables (per natural-log unit increase). The PFAS values were natural-log transformed to reduce the influence of outliers, to improve the model fit, and to make interpretation simpler and more consistent across different PFASs that vary in their ranges of concentration. We also categorized PFAS values into quartiles according to the distribution among controls, using the lowest quartile as the reference group. Moreover, we fitted generalized additive models with a smoothing function of natural-log PFAS values to examine potential nonlinear relations. Five knots were set as the upper limit of number of degrees of freedom, and we compared model fit and visually inspected plots of the smoothed data. We did not find evidence for nonlinearity between natural-log PFAS values and ADHD or autism (data not shown).

Potential confounders were chosen *a priori* considering variables that may influence PFAS exposures and previously suggested risk factors of ADHD or autism. We included maternal age at delivery (≤ 24, 25–29, 30–34, ≥ 35 years), parity (1, > 1), socioeconomic status (low/medium or high), maternal smoking (never, ≤ 9 cigarette/day, > 9 cigarettes/day) and alcohol drinking (yes, no) during pregnancy, mother’s self-reported psychiatric illnesses (yes, no), gestational week of blood draw (4–8, > 8 week), child’s birth year (1998–2000, 2001–2003), and the matching factor child’s sex in the final model. Socioeconomic status was determined based on self-reported maternal and paternal education and occupation using three categories (high, medium, and low): Higher education (4 years beyond high school) or work in management were classified as high; skilled workers and middle-range education as medium; and unskilled workers and unemployed as low (Bech et al. 2005). To determine maternal psychiatric illnesses, we asked women to report whether they had ever seen a doctor or psychologist because of depression, anxiety, childhood psychiatric disorder, family problems/life crisis, or other mental health problems. Additionally, other potential confounders such as father’s age at child’s birth, mother’s prepregnancy body mass index, whether the pregnancy was planned, and season of conception were evaluated but not included in final models because they changed effect estimates of interest minimally (< 1%).

To account for PFAS values below the LLOQ when PFASs were analyzed as continuous variables, we used multiple imputation (Lubin et al. 2004) with the procedure PROC MI in SAS (SAS Institute Inc.) with all six PFASs and all covariates included in the model. Ten simulated complete data sets were generated via imputation, and we employed standard analytical procedures to combine the results (Yuan 2001).

A Pearson correlation matrix for the considered PFASs is presented in Supplemental Material, Table S1. We constructed a “multiple PFAS” model where we simultaneously included all PFASs in one model to examine whether any single PFAS may be of particular importance. We also evaluated potential effect measure modification by child’s sex; we compared the sex-stratified estimates and examined the *p*-value for the PFAS–sex interaction term. For ADHD we also conducted analyses in which we excluded children born after 2000 because the duration of follow-up may not have been long enough to identify children with this diagnosis. For these stratified analyses we used logistic regression without applying sampling weights because the weighted analyses may underestimate uncertainty in our data when the number of actual measured samples is small. Finally, in sensitivity analyses we excluded PFAS values that were greater than three times the 75th percentile (*n* = 2 PFOA, *n* = 7 PFHxS, *n* = 1 PFNA, *n* = 2 PFHpS, *n* = 1 PFDA) to ensure that individuals with extreme exposure values did not disproportionately influence our results. Analyses were performed using SAS version 9.2.

## Results

Table 1 presents the demographic characteristics of cases and controls. Table 2 shows the median and interquartile range distribution of maternal PFAS concentrations during pregnancy in cases and controls.

**Table 1:** Characteristics of study participants [*n* (%)].

Characteristic	ADHD(*n* = 220)	Childhood autism(*n* = 220)	Controls (*n* = 550)
Child’s sex
Male	179 (81.4)	187 (85.0)	440 (80.0)
Female	41 (18.6)	33 (15.0)	110 (20.0)
Mother’s age at delivery (years)
≤ 24	37 (16.8)	28 (12.7)	42 (7.6)
25–29	83 (37.7)	81 (36.8)	235 (42.7)
30–34	72 (32.7)	75 (34.1)	201 (36.5)
≥ 35	28 (12.7)	36 (16.4)	72 (13.1)
Socioeconomic status
Low/medium	112 (50.9)	74 (33.6)	209 (38.0)
High	106 (48.2)	144 (65.5)	339 (61.6)
Parity
1	107 (48.6)	119 (54.1)	247 (44.9)
> 1	100 (45.5)	96 (43.6)	288 (52.4)
Maternal drinking during pregnancy
No	79 (35.9)	79 (35.9)	161 (29.3)
Yes	141 (64.1)	141 (64.1)	389 (70.7)
Maternal smoking during pregnancy
Never	139 (63.2)	142 (64.5)	409 (74.4)
≤ 9 cigarettes/day	32 (14.5)	33 (15.0)	64 (11.6)
> 9 cigarettes/day	49 (22.3)	45 (20.5)	77 (14.0)
Mother’s self-reported psychiatric illnesses
No	167 (75.9)	173 (78.6)	469 (85.3)
Yes	53 (24.1)	47 (21.4)	81 (14.7)
Child’s birth year
1998–2000	133 (60.5)	114 (51.8)	322 (58.5)
2001–2003	87 (39.5)	106 (48.2)	228 (41.5)
Gestational weeks at blood draw
4–8 weeks	87 (39.5)	88 (40.0)	216 (39.3)
> 8 weeks	119 (54.1)	115 (52.3)	305 (55.5)
The missing values for socioeconomic status, parity, and gestational weeks at blood draw are about 1%, 4%, and 7%, respectively.			

**Table 2:** Distribution of maternal plasma PFAS concentrations in cases and controls.

Perfluoroalkyl substance	Abbreviation	Carbon chain length^*a*^	Percent quantifiable in all samples	PFAS concentration (ng/mL) [median (25th, 75th percentile)]^*b*^		
				ADHD (*n* = 215)	Childhood autism (*n* = 213)	Controls (*n* = 545)
Perfluorooctane sulfonate	PFOS	8	100	26.80 (19.20, 35.00)	25.40 (18.73, 32.40)	27.40 (20.40, 35.60)
Perfluorooctanoic acid	PFOA	8	100	4.06 (3.08, 5.50)	3.88 (3.08, 5.28)	4.00 (3.01, 5.42)
Perfluorohexane sulfonate	PFHxS	6	98	0.84 (0.61, 1.15)	0.92 (0.70, 1.17)	0.92 (0.68, 1.23)
Perfluoroheptane sulfonate	PFHpS	7	96	0.30 (0.20, 0.40)	0.28 (0.19, 0.38)	0.30 (0.21, 0.41)
Perfluorononanoic acid	PFNA	9	92	0.42 (0.34, 0.52)	0.41 (0.33, 0.51)	0.43 (0.35, 0.56)
Perfluorodecanoic acid	PFDA	10	90	0.15 (0.11, 0.20)	0.15 (0.11, 0.20)	0.17 (0.12, 0.23)
^***a***^The number of carbons in the fully fluorinated alkyl chain. ^***b***^Concentrations for 17 samples (5 ADHD, 7 autism, and 5 controls) were missing because the samples were either not available from the biobank or failed the extraction process.						

We generally found no association between ADHD or autism in children and PFAS levels in maternal plasma (modeled as natural-log units) (Table 3). We did not detect apparent effect modification by child’s sex (all PFASs and sex interaction *p*-values ≥ 0.25), but because both diagnoses were more prevalent in boys, estimates for girls were less precise (see Supplemental Material, Table S2).

**Table 3:** Risks ratios*^a^* for ADHD and childhood autism in children according to maternal plasma concentrations of PFAS*^b^* in pregnancy.

Prenatal exposure	ADHD^*c*^		Childhood autism^*c*^	
	Adjusted RR^*d*^ (95% CI)	Adjusted RR^*e*^ (95% CI)	Adjusted RR^*d*^ (95% CI)	Adjusted RR^*e*^ (95% CI)
PFOS	0.87 (0.74, 1.02)	1.04 (0.70, 1.56)	0.92 (0.69, 1.22)	1.21 (0.69, 2.13)
PFOA	0.98 (0.82, 1.16)	1.21 (0.84, 1.74)	0.98 (0.73, 1.31)	1.15 (0.68, 1.93)
PFHxS	0.97 (0.88, 1.08)	1.05 (0.91, 1.20)	1.10 (0.92, 1.33)	1.26 (1.00, 1.58)
PFNA	0.80 (0.62, 1.03)	0.99 (0.58, 1.70)	0.80 (0.58, 1.11)	0.84 (0.48, 1.49)
PFHpS	0.91 (0.79, 1.05)	0.93 (0.64, 1.36)	0.91 (0.74, 1.12)	0.82 (0.56, 1.22)
PFDA	0.76 (0.64, 0.91)	0.80 (0.58, 1.11)	0.79 (0.63, 1.01)	0.82 (0.53, 1.28)
^***a***^Inverse probability weights derived from sampling fractions of cases and controls were applied in analyses. ^***b***^Per 1 natural-log unit (ng/mL) increase. ^***c***^We used 215 ADHD cases, 213 autism cases, and 545 controls in analyses. ^***d***^Adjusted for maternal age at delivery, socioeconomic status, parity, smoking and drinking during pregnancy, psychiatric illnesses, gestational week of blood drawn, and child’s sex and birth year. ^***e***^Additionally adjusted including all PFASs in the model.				

When we categorized PFAS values, mothers in the highest quartile of PFOS, PFHxS, PFHpS, and PFDA were less likely to have a child diagnosed with ADHD than mothers in the lowest quartile, after adjustment for potential confounders (Table 4). When all PFASs were simultaneously entered into the model, PFOA and PFNA levels were positively associated with ADHD, whereas negative associations with the other compounds persisted, with most showing monotonic trends. There was some evidence of a positive association between PFHxS and autism, though RRs for the highest quartile were closer to the null than RRs for the second and third quartiles. Similar patterns were found with lower precision of the estimates when we used logistic regression without applying sampling weights (see Supplemental Material, Table S3).

**Table 4:** Risks ratios*^a^* for ADHD and childhood autism in children according to maternal plasma concentrations of PFAS (in quartiles) in pregnancy.

Prenatal exposure^*b*^	ADHD			Childhood autism		
	Crude RR	Adjusted RR^*c*^ (95% CI)	Adjusted RR^*d*^ (95% CI)	Crude RR	Adjusted RR^*c*^ (95% CI)	Adjusted RR^*d*^ (95% CI)
PFOS (ng/mL)
3.85–20.40	1.00	1.00 (reference)	1.00 (reference)	1.00	1.00 (reference)	1.00 (reference)
20.41–27.40	0.83	0.95 (0.79, 1.15)	0.93 (0.75, 1.15)	0.72	0.91 (0.66, 1.25)	1.05 (0.73, 1.50)
27.41–35.60	0.90	0.93 (0.76, 1.13)	0.86 (0.65, 1.12)	0.80	1.01 (0.73, 1.40)	1.20 (0.77, 1.89)
≥ 35.61	0.78	0.79 (0.64, 0.98)	0.65 (0.47, 0.91)	0.60	0.86 (0.59, 1.25)	1.16 (0.65, 2.09)
PFOA (ng/mL)
0.57–3.01	1.00	1.00 (reference)	1.00 (reference)	1.00	1.00 (reference)	1.00 (reference)
3.02–4.00	1.00	1.02 (0.84, 1.23)	1.24 (0.99, 1.55)	1.05	1.13 (0.82, 1.56)	1.11 (0.76, 1.60)
4.01–5.42	1.13	1.09 (0.90, 1.33)	1.46 (1.14, 1.88)	1.03	1.05 (0.74, 1.47)	0.97 (0.63, 1.48)
≥ 5.43	1.07	1.14 (0.92, 1.40)	2.02 (1.49, 2.75)	0.78	0.95 (0.65, 1.38)	0.93 (0.54, 1.59)
PFHxS (ng/mL)
< LLOQ–0.68	1.00	1.00 (reference)	1.00 (reference)	1.00	1.00 (reference)	1.00 (reference)
0.69–0.92	0.97	1.05 (0.88, 1.26)	0.94 (0.76, 1.15)	1.26	1.33 (0.95, 1.87)	1.55 (1.06, 2.28)
0.93–1.23	0.90	0.94 (0.78, 1.14)	0.82 (0.65, 1.02)	1.38	1.50 (1.08, 2.10)	1.86 (1.25, 2.76)
≥ 1.24	0.64	0.67 (0.54, 0.83)	0.56 (0.43, 0.73)	0.94	1.07 (0.73, 1.56)	1.33 (0.84, 2.11)
PFNA (ng/mL)
< LLOQ–0.35	1.00	1.00 (reference)	1.00 (reference)	1.00	1.00 (reference)	1.00 (reference)
0.36–0.43	1.07	1.08 (0.90, 1.30)	1.29 (1.05, 1.59)	1.06	1.06 (0.78, 1.44)	0.94 (0.66, 1.34)
0.44–0.56	1.28	1.12 (0.93, 1.33)	1.48 (1.18, 1.86)	1.03	0.81 (0.59, 1.11)	0.73 (0.49, 1.08)
≥ 0.57	0.75	0.85 (0.69, 1.04)	1.58 (1.17, 2.13)	0.70	0.80 (0.56, 1.12)	0.98 (0.59, 1.63)
PFHpS (ng/mL)
< LLOQ–0.21	1.00	1.00 (reference)	1.00 (reference)	1.00	1.00 (reference)	1.00 (reference)
0.21–0.30	0.74	0.70 (0.58, 0.84)	0.67 (0.54, 0.83)	0.83	0.82 (0.60, 1.12)	0.70 (0.49, 1.01)
0.31–0.41	0.91	0.87 (0.72, 1.05)	0.86 (0.65, 1.13)	0.82	0.92 (0.66, 1.29)	0.83 (0.53, 1.31)
≥ 0.42	0.75	0.71 (0.58, 0.87)	0.81 (0.57, 1.15)	0.66	0.82 (0.57, 1.19)	0.80 (0.44, 1.48)
PFDA (ng/mL)
< LLOQ–0.12	1.00	1.00 (reference)	1.00 (reference)	1.00	1.00 (reference)	1.00 (reference)
0.13–0.17	0.91	0.82 (0.69, 0.97)	0.80 (0.66, 0.96)	1.04	0.93 (0.69, 1.25)	0.99 (0.72, 1.37)
0.18–0.23	0.83	0.87 (0.72, 1.05)	0.91 (0.73, 1.14)	0.98	1.07 (0.77, 1.47)	1.34 (0.92, 1.95)
≥ 0.24	0.51	0.53 (0.43, 0.66)	0.53 (0.40, 0.72)	0.50	0.52 (0.35, 0.77)	0.73 (0.43, 1.24)
^***a***^Inverse probability weights derived from sampling fractions of cases and controls were applied in analyses. ^***b***^PFAS values below the LLOQ were grouped in the lowest quartile. ^***c***^Adjusted for maternal age at delivery, socioeconomic status, parity, smoking and drinking during pregnancy, psychiatric illnesses, gestational week of blood drawn, and child’s sex and birth year. ^***d***^Additionally adjusted including all PFASs in the model.						

Results were similar to those from the primary models when we performed additional sensitivity analyses restricting the analyses to children born before 2001 (see Supplemental Material, Table S4), and excluding extreme PFAS values (results not shown).

## Discussion

Overall, our results do not suggest that prenatal exposure to PFASs increases the risk of ADHD or childhood autism in children. We observed some inverse associations between several PFASs and ADHD after controlling for potential confounders. In the “multiple PFAS” model, we found some positive as well as negative associations between PFASs and ADHD but these might be subject to multicollinearity or sparse data bias. Results were mostly close to null for autism in both single and multiple PFAS models.

Toxicology studies have raised concerns that PFASs are neurotoxic and hormone disruptive and can impair fetal brain development (Johansson et al. 2008; Lau et al. 2003; Long et al. 2013). However, some neurotoxic effects in rats were observed at doses several orders of magnitude higher than the PFAS levels found in the U.S. and Danish general populations (Butenhoff et al. 2009; Fei et al. 2007). Several epidemiologic studies have investigated associations between PFASs and hyperactivity or behavioral problems in children, but the findings have been inconclusive (Braun et al. 2014; Fei and Olsen 2011; Hoffman et al. 2010; Stein and Savitz 2011; Stein et al. 2013). A previous study based on a subset of children from the Danish National Birth Cohort found some inverse associations between prenatal PFOA, but not PFOS, and behavioral problems in 7-year-old children measured by (parent-reported) items in the Strength and Difficulty Questionnaire (Fei and Olsen 2011). Another study also suggested a lower prevalence of ADHD characteristics in children associated with higher estimated *in utero* PFOA exposures based on the Clinical Confidence Index (Stein et al. 2013). There is, however, no biologic explanation for PFASs protecting the developing brain from ADHD, and potential biases such as uncontrolled confounding or selection bias might have driven these unexpected findings. No apparent associations were found between PFASs and autism in current and a previous small study (Braun et al. 2014).

Because several PFASs are moderately to highly correlated, it is difficult to disentangle mixture effects from compound-specific effects. A recent *in vitro* assay reported an additive or more than additive antagonistic effect for a mixture of compounds (PFHxS, PFOS, PFOA, PFNA, and PFDA) on androgen receptor function (Kjeldsen and Bonefeld-Jørgensen 2013). Unfortunately, our sample is too small to allow for interaction analyses between different PFASs. Further experimental studies are needed to determine mechanisms of action for PFAS mixtures on biologic targets that could better inform our population-based studies in terms of the most biologically relevant exposure model to be employed.

It has previously been shown that prenatal exposure to PFASs can increase the incidence of fetal resorption and neonatal deaths in animal models (Abbott et al. 2007; Lau et al. 2007; Luebker et al. 2005). PFASs may interfere with sex and thyroid hormone homeostasis (Kjeldsen and Bonefeld-Jørgensen 2013; Lin et al. 2013; Wang et al. 2014), and it has been suggested that higher PFAS levels are associated with reduced fecundity in women (Buck Louis et al. 2013; Fei et al. 2009) and with an increased risk for miscarriage (Darrow et al. 2014). It is therefore possible that PFAS exposure at a level that reduces fetal or neonatal survival, especially in high-risk fetuses and infants susceptible to neurological disorders such as ADHD and autism, could appear to have null or even protective effects on adverse neurobehavioral outcomes in children based on observational studies, because only live-born children can be followed up and examined.

There are several strengths in our study. First, the PFAS measures were obtained from maternal plasma samples collected in pregnancy before the assessment of the outcomes in the children. Previous studies have shown that PFASs are stable in human serum, and measurements obtained from serum or plasma samples gave comparable results (Ehresman et al. 2007). High correlations between maternal and cord blood PFAS measures were also reported, and these suggested that PFASs measured in maternal plasma can be used as a reasonable surrogate for fetal exposure levels throughout gestations (Fei et al. 2007). Furthermore, the maternal PFAS levels in our study are similar to those previously measured during the same time period in the U.S. general population (Calafat et al. 2007). Study participants were selected from a well-defined nationwide pregnancy cohort with an average of 10.7 years of follow-up, sufficiently long to assess the outcomes of interest. The outcome measures were clinical diagnoses using standardized diagnostic criteria from both the general and psychiatric hospital registries in Denmark, a country with high-quality health care and universal coverage for its population. Diagnoses of childhood autism recorded in the psychiatric registry have previously been shown to have high validity: A study extracted and reviewed the medical records of 499 childhood autism cases from the registry and confirmed the diagnoses for 94% of the cases (Lauritsen et al. 2010). Follow-up was conducted through record linkage that did not require subjects’ responses, thus minimizing chances for selection bias due to subject’s nonresponse.

Our study also has some limitations. Both ADHD and autism are about four times more prevalent in boys, and because of cost limitations we were required to sample no more than 220 cases for each diagnostic group, resulting in few female cases (*n* = 41 with ADHD; *n* = 33 with autism). Thus, our subgroup analyses by sex were relatively imprecise for girls, resulting in effect estimates with wide CIs. For autism, the cases were limited to children diagnosed with childhood autism. Although this is the most severe disorder of the autism spectrum, it constitutes only a part of autistic spectrum disorders; specifically children with Asperger’s syndrome and other pervasive development disorders were not studied. Moreover, we have no data for other endocrine-disrupting chemicals, preventing us from evaluating possible correlations or interactions of PFASs with other ubiquitous environmental chemicals with these properties such as polychlorinated biphenyls (PCBs), organophosphates, bisphenol A, and phthalates (de Cock et al. 2012; Polanska et al. 2012). Further, our blood samples had to be transported to the laboratory by ordinary mail before being processed, which may have induced some random measurement errors.

In summary, we found no consistent evidence that prenatal PFAS exposures were associated with ADHD or childhood autism in children in the Danish National Birth Cohort. Both weak negative associations as well as some positive associations between PFASs and ADHD that we observed in multiple PFAS models should be further explored. We recommend that future studies analyze a larger sample, consider both prenatal exposure and exposure during first year of life, assess the potential mixture effects of exposures to different co-occurring endocrine disruptors, and examine more sensitive indicators such as neuropsychological functioning in children.

## Supplemental Material

(273 KB) PDF

## References

[r1] Abbott BD, Wolf CJ, Schmid JE, Das KP, Zehr RD, Helfant L (2007). Perfluorooctanoic acid–induced developmental toxicity in the mouse is dependent on expression of peroxisome proliferator–activated receptor-alpha.. Toxicol Sci.

[r2] Andersen TF, Madsen M, Jørgensen J, Mellemkjoer L, Olsen JH (1999). The Danish National Hospital Register. A valuable source of data for modern health sciences.. Dan Med Bull.

[r3] Arnold LE (1996). Sex differences in ADHD: conference summary.. J Abnorm Child Psychol.

[r4] Braun JM Kalkbrenner AE Just AC Yolton K Calafat AM Sjödin A 2014 Gestational exposure to endocrine-disrupting chemicals and reciprocal social, repetitive, and stereotypic behaviors in 4- and 5-year-old children: the HOME study. Environ Health Perspect 122 513 520; 10.1289/ehp.1307261 24622245 PMC4014765

[r5] Bech BH, Nohr EA, Vaeth M, Henriksen TB, Olsen J (2005). Coffee and fetal death: a cohort study with prospective data.. Am J Epidemiol.

[r6] Buck RC, Franklin J, Berger U, Conder JM, Cousins IT, de Voogt P (2011). Perfluoroalkyl and polyfluoroalkyl substances in the environment: terminology, classification, and origins.. Integr Environ Assess Manag.

[r7] Buck Louis GM Sundaram R Schisterman EF Sweeney AM Lynch CD Gore-Langton RE 2013 Persistent environmental pollutants and couple fecundity: the LIFE study. Environ Health Perspect 121 231 236; 10.1289/ehp.1205301 23151773 PMC3569685

[r8] Butenhoff JL, Ehresman DJ, Chang SC, Parker GA, Stump DG (2009). Gestational and lactational exposure to potassium perfluorooctanesulfonate (K^+^PFOS) in rats: developmental neurotoxicity.. Reprod toxicol.

[r9] Calafat AM, Kuklenyik Z, Reidy JA, Caudill SP, Tully JS, Needham LL (2007). Serum concentrations of 11 polyfluoroalkyl compounds in the U.S. population: data from the National Health and Nutrition Examination Survey (NHANES) 1999–2000.. Environ Sci Technol.

[r10] Darrow LA, Howards PP, Winquist A, Steenland K (2014). PFOA and PFOS serum levels and miscarriage risk.. Epidemiology.

[r11] de Cock M, Maas YG, van de Bor M (2012). Does perinatal exposure to endocrine disruptors induce autism spectrum and attention deficit hyperactivity disorders? Review.. Acta Paediatr.

[r12] D’eon JC, Mabury SA (2011). Is indirect exposure a significant contributor to the burden of perfluorinated acids observed in humans?. Environ Sci Technol.

[r13] Ehresman DJ, Froehlich JW, Olsen GW, Chang SC, Butenhoff JL (2007). Comparison of human whole blood, plasma, and serum matrices for the determination of perfluorooctanesulfonate (PFOS), perfluorooctanoate (PFOA), and other fluorochemicals.. Environ Res.

[r14] Faraone SV, Sergeant J, Gillberg C, Biederman J (2003). The worldwide prevalence of ADHD: is it an American condition?. World Psychiatry.

[r15] Fei CY, McLaughlin JK, Lipworth L, Olsen J (2009). Maternal levels of perfluorinated chemicals and subfecundity.. Hum Reprod.

[r16] Fei CY McLaughlin JK Tarone RE Olsen J 2007 Perfluorinated chemicals and fetal growth: a study within the Danish National Birth Cohort. Environ Health Perspect 115 1677 1682; 10.1289/ehp.10506 18008003 PMC2072850

[r17] Fei CY Olsen J 2011 Prenatal exposure to perfluorinated chemicals and behavioral or coordination problems at age 7 years. Environ Health Perspect 119 573 578; 10.1289/ehp.1002026 21062688 PMC3080943

[r18] Glynn A, Berger U, Bignert A, Ullah S, Aune M, Lignell S (2012). Perfluorinated alkyl acids in blood serum from primiparous women in Sweden: serial sampling during pregnancy and nursing, and temporal trends 1996–2010.. Environ Sci Technol.

[r19] Gump BB, Wu Q, Dumas AK, Kannan K (2011). Perfluorochemical (PFC) exposure in children: associations with impaired response inhibition.. Environ Sci Technol.

[r20] Hertz-Picciotto I, Delwiche L (2009). The rise in autism and the role of age at diagnosis.. Epidemiology.

[r21] Hoffman K Webster TF Weisskopf MG Weinberg J Vieira VM 2010 Exposure to polyfluoroalkyl chemicals and attention deficit/hyperactivity disorder in U.S. children 12–15 years of age. Environ Health Perspect 118 1762 1767; 10.1289/ehp.1001898 20551004 PMC3002197

[r22] Houde M, Martin JW, Letcher RJ, Solomon KR, Muir DC (2006). Biological monitoring of polyfluoroalkyl substances: a review.. Environ Sci Technol.

[r23] Johansson N, Eriksson P, Viberg H (2009). Neonatal exposure to PFOS and PFOA in mice results in changes in proteins which are important for neuronal growth and synaptogenesis in the developing brain.. Toxicol Sci.

[r24] Johansson N, Fredriksson A, Eriksson P (2008). Neonatal exposure to perfluorooctane sulfonate (PFOS) and perfluorooctanoic acid (PFOA) causes neurobehavioural defects in adult mice.. Neurotoxicology.

[r25] Kato K, Wong LY, Jia LT, Kuklenyik Z, Calafat AM (2011). Trends in exposure to polyfluoroalkyl chemicals in the U.S. population: 1999–2008.. Environ Sci Technol.

[r26] Kjeldsen LS, Bonefeld-Jørgensen EC (2013). Perfluorinated compounds affect the function of sex hormone receptors.. Environ Sci Pollut Res Int.

[r27] Lau C, Anitole K, Hodes C, Lai D, Pfahles-Hutchens A, Seed J (2007). Perfluoroalkyl acids: a review of monitoring and toxicological findings.. Toxicol Sci.

[r28] Lau C, Thibodeaux JR, Hanson RG, Rogers JM, Grey BE, Stanton ME (2003). Exposure to perfluorooctane sulfonate during pregnancy in rat and mouse. II: Postnatal evaluation.. Toxicol Sci.

[r29] Lauritsen MB, Jørgensen M, Madsen KM, Lemcke S, Toft S, Grove J (2010). Validity of childhood autism in the Danish Psychiatric Central Register: findings from a cohort sample born 1990–1999.. J Autism Dev Disord.

[r30] Liew Z, Ritz B, Bonefeld-Jørgensen EC, Henriksen TB, Nohr EA, Bech BH (2014). Prenatal exposure to perfluoroalkyl substances and the risk of congenital cerebral palsy in children.. Am J Epidemiol.

[r31] Lin CY, Wen LL, Lin LY, Wen TW, Lien GW, Hsu SHJ (2013). The associations between serum perfluorinated chemicals and thyroid function in adolescents and young adults.. J Hazard Mater.

[r32] Long M, Ghisari M, Bonefeld-Jørgensen EC (2013). Effects of perfluoroalkyl acids on the function of the thyroid hormone and the aryl hydrocarbon receptor.. Environ Sci Pollut Res Int.

[r33] Lubin JH Colt JS Camann D Davis S Cerhan JR Severson RK 2004 Epidemiologic evaluation of measurement data in the presence of detection limits. Environ Health Perspect 112 1691 1696; 10.1289/ehp.7199 15579415 PMC1253661

[r34] Luebker DJ, York RG, Hansen KJ, Moore JA, Butenhoff JL (2005). Neonatal mortality from in utero exposure to perfluorooctanesulfonate (PFOS) in Sprague-Dawley rats: dose–response, and biochemical and pharamacokinetic parameters.. Toxicology.

[r35] Lyall K Schmidt RJ Hertz-Picciotto I 2014 Maternal lifestyle and environmental risk factors for autism spectrum disorders. Int J Epidemiol 43 443 464; 10.1093/ije/dyt282 24518932 PMC3997376

[r36] Millichap JG (2008). Etiologic classification of attention-deficit/hyperactivity disorder.. Pediatrics.

[r37] Møller LR, Sørensen MJ, Thomsen PH (2007). ICD-10 classification in Danish child and adolescent psychiatry—have diagnoses changed after the introduction of ICD-10?. Nord J Psychiat.

[r38] Munk-Jørgensen P, Kastrup M, Mortensen PB (1993). The Danish Psychiatric Register as a tool in epidemiology.. Acta Psychiatr Scand Suppl.

[r39] Olsen GW Burris JM Ehresman DJ Froehlich JW Seacat AM Butenhoff JL 2007 Half-life of serum elimination of perfluorooctanesulfonate, perfluorohexanesulfonate, and perfluorooctanoate in retired fluorochemical production workers. Environ Health Perspect 115 1298 1305; 10.1289/ehp.10009 17805419 PMC1964923

[r40] Olsen J, Melbye M, Olsen SF, Sørensen TI, Aaby P, Andersen AM (2001). The Danish National Birth Cohort—its background, structure and aim.. Scand J Public Health.

[r41] Pickett J, London E (2005). The neuropathology of autism: a review.. J Neuropathol Exp Neurol.

[r42] Polanczyk G, de Lima MS, Horta BL, Biederman J, Rohde LA (2007). The worldwide prevalence of ADHD: a systematic review and metaregression analysis.. Am J Psychiatry.

[r43] Polanska K, Jurewicz J, Hanke W (2012). Exposure to environmental and lifestyle factors and attention-deficit/hyperactivity disorder in children—a review of epidemiological studies.. Int J Occup Med Environ Health.

[r44] Porterfield SP (2000). Thyroidal dysfunction and environmental chemicals—potential impact on brain development.. Environ Health Perspect.

[r45] Slotkin TA MacKillop EA Melnick RL Thayer KA Seidler FJ 2008 Developmental neurotoxicity of perfluorinated chemicals modeled *in vitro*. Environ Health Perspect 116 716 722; 10.1289/ehp.11253 18560525 PMC2430225

[r46] Statens Serum Institut. (2013). Access to DNBC (Danish National Birth Cohort) data.. http://www.ssi.dk/English/RandD/Research%20areas/Epidemiology/DNBC/For%20researchers.aspx.

[r47] Stein CR Savitz DA 2011 Serum perfluorinated compound concentration and attention deficit/hyperactivity disorder in children 5–18 years of age. Environ Health Perspect 119 1466 1471; 10.1289/ehp.1003538 21665566 PMC3230446

[r48] Stein CR, Savitz DA, Bellinger DC (2013). Perfluorooctanoate and neuropsychological outcomes in children.. Epidemiology.

[r49] Viberg H, Lee I, Eriksson P (2013). Adult dose-dependent behavioral and cognitive disturbances after a single neonatal PFHxS dose.. Toxicology.

[r50] Wang Y Rogan WJ Chen PC Lien GW Chen HY Tseng YC 2014 Association between maternal serum perfluoroalkyl substances during pregnancy and maternal and cord thyroid hormones: Taiwan Maternal and Infant Cohort Study. Environ Health Perspect 122 529 534; 10.1289/ehp.1306925 24577800 PMC4014761

[r51] Yuan Y. (2001). Multiple Imputation for Missing Data: Concepts and New Development. Rockville, MD:SAS Institute Inc.. https://support.sas.com/rnd/app/stat/papers/multipleimputation.pdf.

