# Supplementary material for: Attention Deficit/Hyperactivity Disorder and Childhood Autism in Association with Prenatal Exposure to Perfluoroalkyl Substances: A Nested Case–Control Study in the Danish National Birth Cohort
Source: Environ Health Perspect. 2014 Dec 19;123(4):367–73. doi: 10.1289/ehp.1408412 (PMC4383573; doi:10.1289/ehp.1408412)
Supplement: (273 KB) PDF [file ehp.1408412.s001.508.pdf]

Environmental Health Perspectives doi:10.1289/ehp.1408412

Supplemental Material

Attention Deficit/Hyperactivity Disorder and Childhood Autism in Association with Prenatal Exposure to Perfluoroalkyl Substances: A Nested Case–Control Study in the Danish National Birth Cohort

Zeyan Liew, Beate Ritz, Ondine S. von Ehrenstein, Bodil Hammer Bech, Ellen Aagaard Nohr, Chunyuan Fei, Rossana Bossi, Tine Brink Henriksen, Eva Cecilie Bonefeld-Jørgensen, and Jørn Olsen

Table S1. Pearson correlation coefficients of maternal PFAS concentrations (ng/ml) in controls.

Compound	PFOA	PFHxS	PFNA	PFHpS	PFDA
PFOS	0.71	0.27	0.53	0.86	0.53
PFOA		0.24	0.55	0.73	0.37
PFHxS			0.30	0.36	0.16
PFNA				0.62	0.64
PFHpS					0.51

Table S2. Odds ratios for ADHD and childhood autism in boys and girls according to a 1-ln unit increase in maternal plasma concentrations of PFAS (ng/mL) during pregnancy.

Exposure	ADHD, Boys Adjusted OR[a] (95% CI)	ADHD, Girls Adjusted OR[a] (95% CI)	PFAS x sex interaction p-value	Autism, Boys Adjusted OR[a] (95% CI)	Autism, Girls Adjusted OR[a] (95% CI)	PFAS x sex interaction p-value
PFOS	0.81 (0.52, 1.27)	0.92 (0.30, 2.82)	0.99	0.99 (0.63, 1.55)	0.53 (0.15, 1.86)	0.42
PFOA	0.92 (0.58, 1.45)	1.13 (0.37, 3.48)	0.43	1.07 (0.68, 1.68)	0.52 (0.16, 1.76)	0.37
PFHxS	0.94 (0.72, 1.23)	0.97 (0.38, 2.45)	0.83	1.16 (0.87, 1.54)	0.89 (0.34, 2.35)	0.25
PFNA	0.76 (0.47, 1.22)	1.15 (0.44, 2.98)	0.50	0.85 (0.54, 1.33)	0.62 (0.22, 1.74)	0.58
PFHpS	0.86 (0.63, 1.18)	0.98 (0.42, 2.29)	0.70	0.97 (0.71, 1.33)	0.72 (0.31, 1.69)	0.35
PFDA	0.70 (0.51, 0.96)	1.01 (0.46, 2.24)	0.49	0.85 (0.60, 1.20)	0.52 (0.23, 1.18)	0.32

215 ADHD cases (176 boys, 39 girls), 213 autism cases (180 boys, 33 girls) and 545 controls (435 boys and 110 girls) were used in analyses.

[a]Adjusted for maternal age at delivery, SES, parity, smoking and drinking during pregnancy, psychiatric illnesses, gestational week of blood drawn, and birth year.

Table S3. Odds ratios for ADHD and childhood autism in children according to maternal plasma concentrations of PFAS (in quartiles) in pregnancy.

Prenatal exposure[a]	ADHD No. Cases/ Controls	ADHD Crude OR	ADHD Adjusted OR[b] (95% CI)	ADHD Adjusted OR[c] (95% CI)	Autism No. Cases/ Controls	Autism Crude OR	Autism Adjusted OR[b] (95% CI)	Autism Adjusted OR[c] (95% CI)
**PFOS (ng/ml)**								
3.85 - 20.40	62/140	1.00	1.00 (ref)	1.00 (ref)	69/140	1.00	1.00 (ref)	1.00 (ref)
20.41 - 27.40	53/135	0.92	0.90 (0.57, 1.43)	0.89 (0.52, 1.51)	51/135	0.77	0.87 (0.55, 1.37)	0.92 (0.54, 1.57)
27.41- 35.60	53/136	0.91	0.88 (0.55, 1.41)	0.79 (0.40, 1.54)	53/136	0.79	0.99 (0.62, 1.58)	1.15 (0.60, 2.23)
≥ 35.61	47/134	0.85	0.73 (0.44, 1.20)	0.63 (0.28, 1.42)	40/134	0.61	0.81 (0.48, 1.34)	1.05 (0.45, 2.45)
**PFOA (ng/ml)**								
0.57 - 3.01	49/137	1.00	1.00 (ref)	1.00 (ref)	53/137	1.00	1.00 (ref)	1.00 (ref)
3.02 - 4.00	53/136	1.06	1.02 (0.63, 1.64)	1.28 (0.74, 2.20)	60/136	1.14	1.10 (0.70, 1.74)	1.13 (0.66, 1.92)
4.01 - 5.42	56/136	1.11	1.09 (0.67, 1.76)	1.54 (0.82, 2.87)	55/136	1.05	1.04 (0.65, 1.68)	1.02 (0.56, 1.88)
≥ 5.43	57/136	1.12	1.11 (0.66, 1.86)	2.02 (0.95, 4.27)	45/136	0.86	0.92 (0.54, 1.54)	0.94 (0.43, 2.03)
**PFHxS (ng/ml)**								
<LLOQ - 0.68	63/141	1.00	1.00 (ref)	1.00 (ref)	48/141	1.00	1.00 (ref)	1.00 (ref)
0.69 - 0.92	60/136	0.99	0.96 (0.61, 1.50)	0.85 (0.52, 1.39)	59/136	1.27	1.33 (0.83, 2.11)	1.44 (0.86, 2.39)
0.93 - 1.23	53/133	0.92	0.86 (0.54, 1.36)	0.75 (0.44, 1.29)	62/133	1.37	1.47 (0.92, 2.35)	1.80 (1.04, 3.10)
≥ 1.24	39/135	0.73	0.60 (0.36, 1.00)	0.54 (0.29, 1.01)	44/135	0.96	1.03 (0.62, 1.72)	1.30 (0.70, 2.43)
**PFNA (ng/ml)**								
<LLOQ - 0.35	62/149	1.00	1.00 (ref)	1.00 (ref)	66/149	1.00	1.00 (ref)	1.00 (ref)
0.36 - 0.43	53/127	1.00	1.05 (0.67, 1.66)	1.23 (0.75, 2.02)	56/127	1.00	1.08 (0.69, 1.68)	0.99 (0.60, 1.62)
0.43 - 0.56	60/138	1.03	1.15 (0.74, 1.78)	1.57 (0.91, 2.70)	51/138	0.83	0.82 (0.52, 1.28)	0.76 (0.43, 1.32)
≥ 0.57	40/131	0.80	0.82 (0.50, 1.33)	1.52 (0.77, 3.03)	40/131	0.69	0.78 (0.49, 1.26)	0.88 (0.43, 1.80)
**PFHpS (ng/ml)**								
<LLOQ - 0.21	65/140	1.00	1.00 (ref)	1.00 (ref)	66/140	1.00	1.00 (ref)	1.00 (ref)
0.21 - 0.30	48/141	0.80	0.71 (0.45, 1.12)	0.70 (0.40, 1.21)	55/141	0.83	0.83 (0.53, 1.30)	0.76 (0.45, 1.30)
0.30 - 0.41	54/129	0.93	0.84 (0.53, 1.34)	0.87 (0.44, 1.73)	49/129	0.81	0.90 (0.56, 1.43)	0.83 (0.42, 1.61)
≥ 0.42	48/135	0.83	0.67 (0.41, 1.11)	0.82 (0.34, 1.99)	43/135	0.68	0.80 (0.49, 1.32)	0.84 (0.35, 2.03)
**PFDA (ng/ml)**								
<LLOQ - 0.12	70/139	1.00	1.00 (ref)	1.00 (ref)	64/139	1.00	1.00 (ref)	1.00 (ref)
0.13 - 0.17	66/159	0.88	0.77 (0.50, 1.18)	0.79 (0.50, 1.26)	69/159	0.94	0.92 (0.60, 1.41)	1.00 (0.64, 1.58)
0.18 - 0.23	49/121	0.86	0.93 (0.58, 1.48)	1.01 (0.58, 1.78)	53/121	0.95	1.08 (0.68, 1.71)	1.40 (0.80, 2.45)
≥ 0.24	30/126	0.57	0.52 (0.31, 0.87)	0.59 (0.29, 1.18)	27/126	0.47	0.52 (0.30, 0.88)	0.77 (0.37, 1.60)

[a]PFAS values below the lower limit of quantitation (LLOQ) were grouped in the lowest quartile. [b]Adjusted for maternal age at delivery, SES, parity, smoking and drinking during pregnancy, psychiatric illnesses, gestational week of blood drawn, child's sex and birth year. [c]Adjusted for all covariates in b) additionally including all PFASs in the model.

Table S4. Odds ratios[a] for ADHD and childhood autism according to a 1-ln unit increase in maternal plasma concentrations of PFAS in pregnancy (ng/mL), among children born 1998-2000.

Prenatal exposure	ADHD Adjusted OR[b] (95% CI)	ADHD Adjusted OR[c] (95% CI)
PFOS	0.62 (0.36, 1.06)	0.71 (0.22, 2.29)
PFOA	0.95 (0.54, 1.66)	1.74 (0.68, 4.44)
PFHxS	0.92 (0.68, 1.23)	1.02 (0.72, 1.44)
PFNA	0.65 (0.37, 1.12)	0.96 (0.44, 2.10)
PFHpS	0.69 (0.45, 1.07)	0.82 (0.31, 2.14)
PFDA	0.62 (0.42, 0.90)	0.77 (0.44, 1.33)

[a]129 ADHD cases, 109 autism cases and 317 controls were used in analyses. [b]Adjusted for maternal age at delivery, SES, parity, smoking and drinking during pregnancy, mother's psychiatric illnesses, gestational week of blood drawn, child's sex, and child's birth year. [c]Adjusted for all covariates in b) additionally including all PFASs in the model.
